# UAV Control on the Basis of 3D Landmark Bearing-Only Observations

**DOI:** 10.3390/s151229768

**Published:** 2015-11-27

**Authors:** Simon Karpenko, Ivan Konovalenko, Alexander Miller, Boris Miller, Dmitry Nikolaev

**Affiliations:** Institute for Information Transmission Problems RAS, Bolshoy Karetny per. 19, Build. 1, GSP-4, Moscow 127051, Russia; simon.karpenko@gmail.com (S.K.); konovalenko@iitp.ru (I.K.); amiller@iitp.ru (A.M.); dimonstr@iitp.ru (D.N.)

**Keywords:** UAV, visual odometry, projective geometry, video stream, feature points, modified Kalman filter, control

## Abstract

The article presents an approach to the control of a UAV on the basis of 3D landmark observations. The novelty of the work is the usage of the 3D RANSAC algorithm developed on the basis of the landmarks’ position prediction with the aid of a modified Kalman-type filter. Modification of the filter based on the pseudo-measurements approach permits obtaining unbiased UAV position estimation with quadratic error characteristics. Modeling of UAV flight on the basis of the suggested algorithm shows good performance, even under significant external perturbations.

## 1. Introduction

Modern UAV’s navigation systems use the standard elements of INS (inertial navigation systems) along with GPS, which permits correcting the bias and improving the UAV localization, which are necessary for resolving mapping issues, targeting and reconnaissance tasks [1]. The use of computer vision as a secondary or a primary method for autonomous navigation of UAVs has been discussed frequently in recent years, since the classical combination of GPS and INS systems cannot sustain autonomous flight in many situations [2]. UAV autonomous missions usually need so-called data fusion, which is a difficult task, especially for standard INS and vision equipment. It is clear that cameras provide visual information in a different form, inapplicable to UAV direct control, and therefore, one needs an additional on-board memory and special recognition algorithms.

### 1.1. Visual-Based Navigation Approaches

Several studies have demonstrated the effectiveness of approaches based on motion field estimation and feature tracking for visual odometry [3]. Vision-based methods have been proposed even in the context of autonomous landing management [2]. In [4], visual odometry based on geometric homography was proposed. However, the homography analysis uses only 2D reference points coordinates, though for the evaluation of the current UAV altitude, the 3D coordinates are necessary. All such approaches presume the presence of some recognition system in order to detect the objects nominated in advance. Examples of such objects can be special buildings, crossroads, tops of mountains, and so on. The principal difficulties are the different scale and aspect angles of observed and stored images, which leads to the necessity of huge template libraries in the memory of the UAV control system. Here, one can avoid this difficulty, because of the usage of another approach based on the observation of so-called feature points [5] that are scale and aspect angle invariant. For this purpose, the technology of feature points [6] is used. In [7], the approach based on the coordinate correspondence of the reference points observed by the on-board camera and the reference points on the map loaded into the UAV’s memory before the mission start had been suggested. During the flight, these maps are compared to the frame of the land, directly observed with the help of an on-board video camera. As a result, one can detect the current location and orientation without time-error accumulation. These methods are invariant to some transformations, and they are noise-stable, so that predetermined maps can be different in scale, aspect angle, season, luminosity, weather conditions, *etc*. This technology appeared in [8]. The contribution of this work is the usage of a modified unbiased pseudo-measurements filter for bearing-only observations of some reference points with known terrain coordinates.

### 1.2. Kalman Filter

In order to obtain metric data from visual observations, one needs first to make observations from different positions *i.e.*, *triangulation* and then use nonlinear filtering. However, all nonlinear filters either have unknown bias [9] or are very difficult for on-board implementation, like the Bayesian-type estimation [10,11]. Approaches for position estimation based on bearing-only observations had been analyzed long ago, especially for submarine applications [12] and nowadays for UAV applications [1].

A comparison of different nonlinear filters for bearing-only observations in the issue of ground-based object localization [13] shows that the EKF (extended Kalman filter), the unscented Kalman filter, the particle filter and the pseudo-measurement filter give almost the same level of accuracy, while the pseudo-measurement filter is usually more stable and simple for on-board implementation. This observation is in accordance with older results [12], where all of these filters were compared in the issue of moving object localization. It has been mentioned that all of these filters have bias, which makes their use in data fusion issues rather problematic [14]. The principle requirement for such filters in data fusion is the non-biased estimate with the known mean square characterization of the error. Among the variety of possible filters, the pseudo-measurement filter can be easily modified to satisfy the data fusion demands. The idea of such nonlinear filtering was developed by V.S. Pugachev and I. Sinitsyn in the form of so-called conditionally-optimal filtering [15], which provides the non-biased estimation within the class of linear filters with the minimum mean squared error. In this paper, we develop such a filter (the so-called pseudo-measurement Kalman filter (PKF)) for the UAV position estimation and give the algorithm for path planning along with the reference trajectory under external perturbations and noisy measurements.

### 1.3. Optical Absolute Positioning

Some known aerospace maps of a terrain in a flight zone are loaded into the aircraft memory before the start of a flight. During the flight, these maps are compared to the frame of the land, directly observed with the help of an on-board video camera. For this purpose, the technology of feature points [6] is used. As a result, one can detect the current location and orientation without time-error accumulation. These methods are invariant to some transformations and are also noise-stable, so that predetermined maps can vary in height, season, luminosity, weather conditions, *etc*. Furthermore, from the moment of the previous plane surveying, the picture of this landscape can be changed due to human and natural activity. All approaches based on the capturing of the objects assigned in advance presume the presence of some on-board recognition system in order to detect and recognize such objects. Here, we avoid this difficulty by using the observation of feature points [5] that are scale and aspect angle invariant. In addition, the modified pseudo-measurements Kalman filtering (PKF) is used for the estimation of UAV positions and the control algorithm.

### 1.4. Outline of the Approach and the Article Structure

One of the principal parts of this research is an approach to the estimation of the UAV position on the basis of the bearing-only observations. The original filter that uses the idea of pseudo-measurements had been suggested in reference [16] for the case of the azimuth bearing of the terrain objects nominated in advance. In reference [17], this approach had been extended to the case of two angle measurements, namely azimuth and elevation. However, the usage of this approach as a real navigation tool needs huge on-board memory and a sophisticated recognition algorithm, since the template and in-flight observed images, even of the same object, are rather different due to the changes of illuminance, the altitude of flight and the aspect angles. That is why the method based on the observation of feature points looks more attractive for in-flight implementation. In reference [7], an algorithm joining together the feature points approach and modified PKF had been suggested, though for 2D feature point localization, while the more advanced 3D localization had been suggested in references [18,19], which are the shortened versions of the methodology presented in this article.

In this work, we use a computer simulation of a UAV flight and on-board video camera imaging. The simulation program is written in MATLAB. The type of feature points is ASIFT, realized in OpenCV (Python) [20]. Feature points in this model work as in a real flight, because the images for the camera model and for the template images were transformed by projective mapping and created by observations from different satellites.

The next section presents the original RANSAC algorithm for 3D feature point localization. Section 3 and Section 4 give the description of PKF, providing the unbiased estimation of the UAV position with the estimate of quadratic errors. Section 5 describes the locally optimal control algorithm for tracking the reference trajectory on the basis of PKF estimation of the UAV position. In Section 6, we give a new approach to the RANSAC robustness with the use of the UAV motion model. Section 7 presents the modeling results, and Section 8 is the conclusions.

## 2. Random Sample Consensus for Isometry

**Figure 1:**
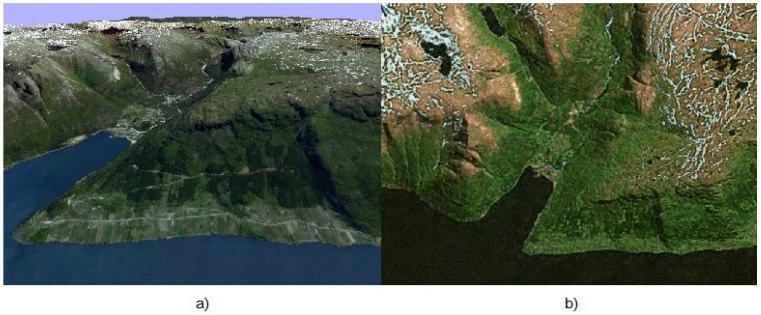
(**a**) Image $I_{c}$ of the on-board camera of the UAV; (**b**) template image loaded in the UAV memory in advance.

At every step, the algorithm deals with two images of a 3D landscape. An example of the landscape used for modeling is shown in Figure 1.

**Figure 2:**
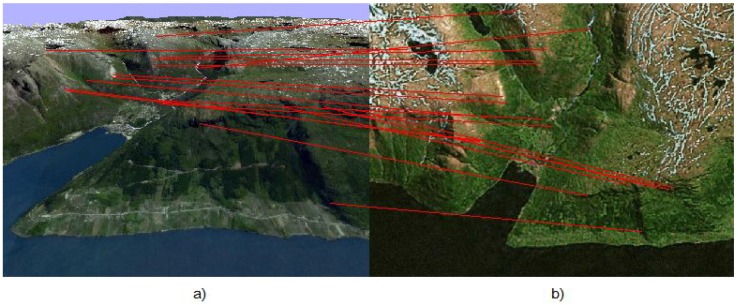
(**a**) Image $I_{c}$ of the on-board camera of the UAV; (**b**) template image loaded in the UAV memory in advance.

The first image $I_{c}$ is obtained from the on-board UAV camera, the position of which is unknown and should be estimated. The second image $I_{m}$ was taken previously from a known position and uploaded into the UAV memory. The ASIFT method is used for both images to detect feature points, which are specified in pixels:

$$c_{i}=\left|\begin{array}{c} c_{xi}\\ c_{yi} \end{array}\right|$$

and:

$$m_{i}=\left|\begin{array}{c} m_{xi}\\ m_{yi} \end{array}\right|$$

and calculates their descriptors. The correspondence between images is constructed by using these descriptors, and thereby, the feature points are combined in pairs. However, many of these pairs are wrong, and therefore, these pairs are considered as outliers or they are not. The result of ASIFT correspondence is shown in Figure 2.

The Earth coordinate system is the Cartesian coordinate system, which is rigidly connected with the Earth. Therefore, the algorithm uses a 3D terrain map of the area from which the image $I_{m}$ was taken and over which the UAV flies. Therefore, one can determine the coordinates of the points:

$$r_{i}=\left|\begin{array}{c} x_{i}\\ y_{i}\\ z_{i} \end{array}\right|$$

which generated $m_{i}$ points in the Earth coordinate system. However, if *i* corresponds to the pair of points that is not an outlier, then the point $r_{i}$ also generates a point $c_{i}$ in the UAV camera.

Another Cartesian coordinate system is rigidly connected with the UAV camera. The axis of the camera is parallel to the axis *z*. The transformation from the Earth coordinate system to the UAV coordinate system has the form:

$$r^{\prime }=A\left(r-b\right)$$

where *b* represents the coordinates of the camera in the Earth coordinate system and *A* is the orthogonal ($AA^{T}=I$) rotation matrix defining the orientation of the UAV camera. Then, the points $r_{i}$ in the camera coordinate system are:

$$r_{i}^{\prime }=A\left(r_{i}-b\right)$$

To define the relation between $r_{i}$ and the feature points $c_{i}$, one can use the model of the camera obscura. This model gives a central projection on the plane:

$$c_{i}=\left|\begin{array}{c} \frac{\rho_{xi}}{\rho_{zi}}\\ \frac{\rho_{yi}}{\rho_{zi}} \end{array}\right|$$

where:

$$\left|\begin{array}{c} \rho_{xi}\\ \rho_{yi}\\ \rho_{zi} \end{array}\right|=\rho_{i}=Kr_{i}^{\prime }=KA\left(r_{i}-b\right)$$

where *K* is a known calibration matrix of the camera.

Thus, the task is to estimate *A* and *b* on the basis of known $c_{i},$ $r_{i},$ K. The minimum number of feature point pairs needed to solve this task is three.

One can give the solution of the problem under the assumption that there are just three pairs:

i={1,2,3}

Points $r_{i}^{\prime }$ form a triangle in the space with the following sides:

$$\rho_{1}=\left|\right|r_{2}^{\prime }-r_{3}^{\prime }{\left|\right|}_{2},\rho_{2}=\left|\right|r_{3}^{\prime }-r_{1}^{\prime }{\left|\right|}_{2},\rho_{3}=\left|\right|r_{1}^{\prime }-r_{2}^{\prime }{\left|\right|}_{2}$$

Meanwhile, due to the rectilinear propagation of light, each point $r_{i}^{\prime }$ lies on the beam $r^{\prime }=a_{i}t$, where *t* is a scalar parameter, and:

$$a_{i}=K^{-1}\left|\begin{array}{c} c_{xi}\\ c_{yi}\\ 1 \end{array}\right|$$

In order to find $r_{i}^{\prime }$, we have to determine parameters $t_{i},$ i={1,2,3} that satisfy the system of quadratic equations:

$$\left\{\begin{array}{c} {\left(a_{2}t_{2}-a_{3}t_{3}\right)}^{T}\left(a_{2}t_{2}-a_{3}t_{3}\right)=\rho_{1}^{2}\\ {\left(a_{3}t_{3}-a_{1}t_{1}\right)}^{T}\left(a_{3}t_{3}-a_{1}t_{1}\right)=\rho_{2}^{2}\\ {\left(a_{1}t_{1}-a_{2}t_{2}\right)}^{T}\left(a_{1}t_{1}-a_{2}t_{2}\right)=\rho_{3}^{2} \end{array}\right.$$

For the given $t_{1}$, this system may be either solved analytically or has no solution. A determination of $t_{1}$ can be done numerically, for example by the bisection method.

Finally, one can obtain the coordinates of three points on the Earth’s surface in the camera coordinate system $r_{i}^{\prime }$ and, at the same time, in the Earth coordinate system $r_{i}$. The connection between them is: $r_{i}^{\prime }=A\left(r_{i}-b\right)$. Since *A* is the orthogonal matrix, then y=Ax implies ${\left||y|\right|}_{2}={\left||x|\right|}_{2}$; thereby:

$$r_{i}^{\prime T}r_{i}^{\prime }={\left(r_{i}-b\right)}^{T}\left(r_{i}-b\right)$$

Therefore, we have eliminated *A* and obtained the problem of finding the intersection of three spheres, which can be solved analytically. This problem may have two solutions; one of them will be rejected later. When *b* has been found, solutions for *A* are as follows:

$$A=\left|\begin{array}{ccc} r_{1}^{\prime } & r_{2}^{\prime } & r_{3}^{\prime } \end{array}\right|{\left|\begin{array}{ccc} r_{1}-b & r_{2}-b & r_{3}-b \end{array}\right|}^{-1}$$

Therefore, there are two options, and only one of them is correct:

$$\begin{array}{c} \left|\begin{array}{c} a_{11}\\ a_{21}\\ a_{31} \end{array}\right|=\left|\begin{array}{c} a_{12}\\ a_{22}\\ a_{32} \end{array}\right|\times \left|\begin{array}{c} a_{13}\\ a_{23}\\ a_{33} \end{array}\right|\;\;\mathrm{or}\;\left|\begin{array}{c} a_{11}\\ a_{21}\\ a_{31} \end{array}\right|=-\left|\begin{array}{c} a_{12}\\ a_{22}\\ a_{32} \end{array}\right|\times \left|\begin{array}{c} a_{13}\\ a_{23}\\ a_{33} \end{array}\right| \end{array}$$

If the first one is correct, then the second one corresponds exactly to the turnover.

As a result, *A* and *b* have been found by using three pairs of feature points and the height map. However, this approach alone is not suitable as the final solution, due to the following problems:

1. The method gives either knowingly false solution or no solution at all if among the three points there are outliers.
2. There is a strong dependence on the noise in the feature points’ location.

Both problems may be solved with random sample consensus (RANSAC) [21,22]. From the general selection of points, one needs to select *N* times a subsample of size three. For each subsample j={1,2,⋯,N}, one can calculate $A_{j}$ and $b_{j}$, which allows one to simulate the generation of all feature points on the UAV camera:

$$c_{ji}=\left|\begin{array}{c} \frac{\rho_{jxi}}{\rho_{jzi}}\\ \frac{\rho_{jyi}}{\rho_{jzi}} \end{array}\right|,\;\;\;\rho_{j}=Kr_{ji}^{\prime }=KA_{j}\left(r_{ji}-b_{j}\right)$$

Then, one can evaluate which points are the outliers by the threshold $s_{ji}=1(||c_{i}-c_{ji}{\left|\right|}_{2}<d)$, where *d* is the threshold. Here, $s_{ji}=0$ means that projection of the *i*-th point on the *j*-th point is counted as an outlier, otherwise $s_{ji}=1.$ The answer will be the following:

$$A=A_{J},\;\;\;b=b_{J},\;\;\;J=\arg\max_{j}\sum_{i}s_{ji}$$

Therefore, we really solve the problem of outliers. Next, we find the required number of *N* subsamples of a size of three, such that among them, there will be at least one subsample without outliers with probability p. Let the proportion of outliers be 1-w. It is easy to see that [21]:

$$N\left(p\right)=\frac{\log(1-p)}{\log(1-w^{3})}$$

In the case when $w=\frac{1}{2}$: N(0.9999)≈69, which shows the high efficiency of algorithm. After that, the points marked as outliers are removed from consideration. The clarification of the response is made by the numerical solution of the following optimization problem on the set of remaining points:

$$\left\{A^{*},b^{*}\right\}=\arg\min_{A,b}\sum_{i}\left|\right|c_{i}-c_{i}\left(A,b\right){\left|\right|}_{2}^{2}$$

Thereby, the second problem of noise reduction may be solved. However, one can use a more advanced procedure, which takes into account the motion model. A more stable solution may be obtained with the aid of so-called robust RANSAC [23]; the idea is to use predicted values of (A,b) for the preliminary rejection of outliers from the pairs of observed feature points. Therefore, if on the *k*-th step of the filtering procedure, the values $\left(A_{k},b_{k}\right)$ have been obtained, one can use the following values on the (k+1)-th step:

(1) $$\left(A_{k+1},b_{k+1}\right)=\left(A_{k},{\hat{b}}_{k+1}\right)$$

where ${\hat{b}}_{k+1}$ is the predicted estimate of the UAV attitude obtained on the basis of the PKF estimate. The filtering approach is described in Section 5.

## 3. Filtering Problem Statement

The problem of bearing-only filtering is considered to determine the coordinates of the UAV, which can observe some objects with known coordinates. These objects can be either the well recognizable objects or a network of radio-beacon stations with a well-specified frequency and known coordinates. In this work, the function of beacons is performed by the feature points determined with the aid of the RANSAC algorithm. The UAV has the standard set of INS devices, which enables it to perform the flight with some degree of accuracy, which, however, is not sufficient for mission completion.

### 3.1. Model of the UAV’s Motion

We assume that a UAV motion is described by three coordinates $\left(X\left(t_{k}\right),Y\left(t_{k}\right),Z\left(t_{k}\right)\right)$ and velocities $(V_{x}\left(t_{k}\right),V_{y}\left(t_{k}\right),V_{z}\left(t_{k}\right)).$ At times $t_{k}=k\Delta t,\;k=1,2,...$, these coordinates satisfy the following equations:

(2) $$X\left(t_{k+1}\right)=FX\left(t_{k}\right)+Ba\left(t_{k}\right)+W\left(t_{k}\right)$$

where:

$$X\left(t_{k}\right)={\left(X\left(t_{k}\right),Y\left(t_{k}\right),Z\left(t_{k}\right),V_{x}\left(t_{k}\right),V_{y}\left(t_{k}\right),V_{z}\left(t_{k}\right)\right)}^{T}$$

is the vector of state-velocities,

$$a\left(t_{k}\right)={\left(a_{x}\left(t_{k}\right),a_{y}\left(t_{k}\right),a_{z}\left(t_{k}\right)\right)}^{T}$$

is the vector of accelerations, which we consider as controls,

$$W\left(t_{k}\right)={\left(0,0,0,W_{x}\left(t_{k}\right),W_{y}\left(t_{k}\right),W_{z}\left(t_{k}\right)\right)}^{T}$$

is the vector of current perturbations, modeling the turbulence components of the wind and the autopilot errors, as well. The matrices *A* and *B* are equal:

$$F=\left(\begin{array}{cccccc} 1 & 0 & 0 & \Delta t & 0 & 0\\ 0 & 1 & 0 & 0 & \Delta t & 0\\ 0 & 0 & 1 & 0 & 0 & \Delta t\\ 0 & 0 & 0 & 1 & 0 & 0\\ 0 & 0 & 0 & 0 & 1 & 0\\ 0 & 0 & 0 & 0 & 0 & 1 \end{array}\right)$$

$$B=\left(\begin{array}{ccc} \frac{\Delta t^{2}}{2} & 0 & 0\\ 0 & \frac{\Delta t^{2}}{2} & 0\\ 0 & 0 & \frac{\Delta t^{2}}{2}\\ \Delta t & 0 & 0\\ 0 & \Delta t & 0\\ 0 & 0 & \Delta t \end{array}\right)$$

and stochastic Equation (2) describes a controlled and perturbed UAV motion.

### 3.2. Measurements

**Figure 3:**
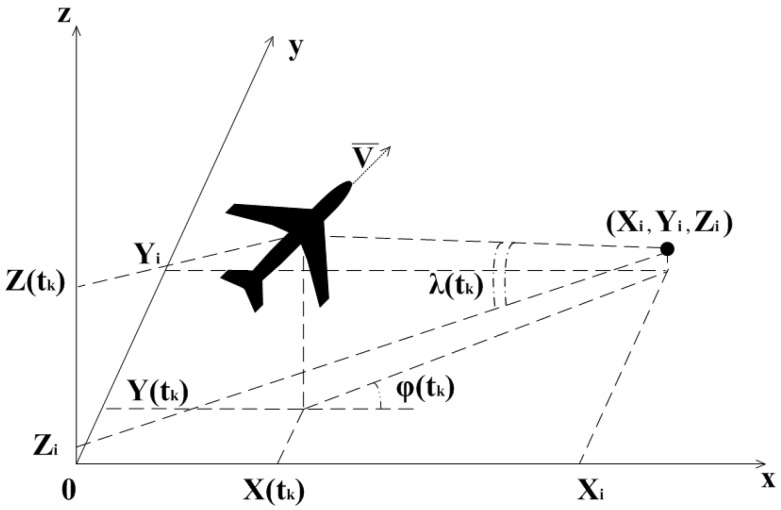
Scheme of the measurements of bearing angles. $X_{i},Y_{i},Z_{i}$ are the coordinates of the *i*-th feature point; $\lambda \left(t_{k}\right),\phi \left(t_{k}\right)$ are the elevation and azimuth bearing angles, measured at the moment $t_{k}.$

Assume that $\left(X_{i},Y_{i},Z_{i}\right)$ are the coordinates of the *i*-th reference point and $\phi_{i}\left(t_{k}\right),\lambda_{i}\left(t_{k}\right)$ are the bearing angles on that point. The measuring scheme is shown in Figure 3.

At moment $t_{k}$, these angles satisfy the relations:

(3) $$\begin{array}{c} \frac{Y_{i}+\varepsilon_{k}^{y}-Y\left(t_{k}\right)}{X_{i}+\varepsilon_{k}^{x}-X\left(t_{k}\right)}I_{i}\left(t_{k}\right)=\tan\phi_{i}\left(t_{k}\right)+\varepsilon_{k}^{\phi }\\ \frac{Z_{i}+\varepsilon_{k}^{z}-Z\left(t_{k}\right)}{\sqrt{{\left(X_{i}+\varepsilon_{k}^{x}-X\left(t_{k}\right)\right)}^{2}+{\left(Y_{i}+\varepsilon_{k}^{y}-Y\left(t_{k}\right)\right)}^{2}}}I_{i}\left(t_{k}\right)=\tan\lambda_{i}\left(t_{k}\right)+\varepsilon_{k}^{\lambda } \end{array}$$

where $\varepsilon_{k}^{x}∼\mathrm{WN}\left(0,\sigma_{x}^{2}\right),$ $\varepsilon_{k}^{y}∼\mathrm{WN}\left(0,\sigma_{y}^{2}\right),$ $\varepsilon_{k}^{z}∼\mathrm{WN}\left(0,\sigma_{z}^{2}\right),$ $\varepsilon_{k}^{\phi }∼\mathrm{WN}\left(0,\sigma_{\phi }^{2}\right),$ $\;\varepsilon_{k}^{\lambda }∼\mathrm{WN}\left(0,\sigma_{\lambda }^{2}\right)$ are uncorrelated random variables with zero means and variances $\sigma_{x}^{2},$ $\sigma_{y}^{2},$ $\sigma_{z}^{2},$ $\sigma_{\phi }^{2},\sigma_{\lambda }^{2}$, defined as errors in the measurement of the coordinates of the *i*-th reference point and of the tangents of angles $\phi_{i}\left(t_{k}\right),\lambda_{i}\left(t_{k}\right)$ and forming the white noise sequences.

**Remark 1.**  In the majority of works based on the method of pseudo-measurements, another model is used. It assumes the measurements of the angles with Gaussian errors (see [12,14] and most of the successive works). However, in the real definition of the object position in the image or in the matrix of sensors, the system measures the distance between the object image and the center of the sensor, that is the tangent of the bearing angle. This simple observation allows one to find the unbiased estimate of the UAV coordinates.

One can rewrite Equation (3) for angle $\lambda_{i}\left(t_{k}\right)$ as follows:

(4) $$\frac{Z_{i}+\varepsilon_{k}^{z}-Z\left(t_{k}\right)}{Y_{i}+\varepsilon_{k}^{y}-Y\left(t_{k}\right)}\sin\phi_{i}\left(t_{k}\right)I_{i}\left(t_{k}\right)=\frac{\sin\lambda_{i}\left(t_{k}\right)}{\cos\lambda_{i}\left(t_{k}\right)}+\varepsilon_{k}^{\lambda }$$

**Remark 2.**  The indicator function $I_{i}\left(t_{k}\right)=1$ if at $t_{k}$ the bearing of the i-th reference point occurs, and $I_{i}\left(t_{k}\right)=0$ otherwise. For convenience, we assume that $I_{i}\left(t_{k}\right)=1.$

Therefore, at the moment $t_{k}$, the UAV control system determines the angles $\phi_{i}\left(t_{k}\right)$ and $\lambda_{i}\left(t_{k}\right)$, related to the coordinates of the UAV $\left(X\left(t_{k}\right),Y\left(t_{k}\right),Z\left(t_{k}\right)\right)$ as follows:

(5) $$\begin{array}{c} \left(Y_{i}+\varepsilon_{k}^{y}-Y\left(t_{k}\right)\right)\cos\phi_{i}\left(t_{k}\right)-\left(X_{i}+\varepsilon_{k}^{x}-X\left(t_{k}\right)\right)\sin\phi_{i}\left(t_{k}\right)\\ =\varepsilon_{k}^{\phi }\left(X_{i}+\varepsilon_{k}^{x}-X\left(t_{k}\right)\right)\cos\phi_{i}\left(t_{k}\right)\\ \left(Z_{i}+\varepsilon_{k}^{z}-Z\left(t_{k}\right)\right)\sin\phi_{i}\left(t_{k}\right)\cos\lambda_{i}\left(t_{k}\right)-\left(Y_{i}+\varepsilon_{k}^{y}-Y\left(t_{k}\right)\right)\sin\lambda_{i}\left(t_{k}\right)\\ =\varepsilon_{k}^{\lambda }\left(Y_{i}+\varepsilon_{k}^{y}-Y\left(t_{k}\right)\right)\cos\lambda_{i}\left(t_{k}\right) \end{array}$$

## 4. Modified Kalman Filtering on the Basis of Pseudo-Measurements

### 4.1. Linear Measurements Model

The idea of the pseudo-measurement method is to separate in Equation (5) the observable and non-observable values, which gives the following observation equations:

(6) $$\begin{array}{c} m_{k}^{\phi }=Y_{i}\cos\phi_{i}\left(t_{k}\right)-X_{i}\sin\phi_{i}\left(t_{k}\right)=Y\left(t_{k}\right)\cos\phi_{i}\left(t_{k}\right)-X\left(t_{k}\right)\sin\phi_{i}\left(t_{k}\right)\\ -\varepsilon_{k}^{y}\cos\phi_{i}\left(t_{k}\right)+\varepsilon_{k}^{x}\sin\phi_{i}\left(t_{k}\right)+\varepsilon_{k}^{\phi }\left(X_{i}+\varepsilon_{k}^{x}-X\left(t_{k}\right)\right)\cos\phi_{i}\left(t_{k}\right)\\ m_{k}^{\lambda }=Z_{i}\sin\phi_{i}\left(t_{k}\right)\cos\lambda_{i}\left(t_{k}\right)-Y_{i}\sin\lambda_{i}\left(t_{k}\right)=Z\left(t_{k}\right)\sin\phi_{i}\left(t_{k}\right)\cos\lambda_{i}\left(t_{k}\right)-Y\left(t_{k}\right)\sin\lambda_{i}\left(t_{k}\right)\\ -\varepsilon_{k}^{z}\sin\phi_{i}\left(t_{k}\right)\cos\lambda_{i}\left(t_{k}\right)+\varepsilon_{k}^{y}\sin\lambda_{i}\left(t_{k}\right)+\varepsilon_{k}^{\lambda }\left(Y_{i}+\varepsilon_{k}^{y}-Y\left(t_{k}\right)\right)\cos\lambda_{i}\left(t_{k}\right) \end{array}$$

where $X_{i},Y_{i},Z_{i}$ represent the coordinates of the *i*-th feature point determined with the aid of the RANSAC algorithm and $\phi_{i},\lambda_{i}$ are the corresponding observable bearing angles measured by the system. Thus, the left-hand side of Equation (6), that is $\left(m_{k}^{\phi },m_{k}^{\lambda }\right)$, corresponds to the observable values, whereas the right-hand side containing the coordinates of the UAV corresponds to the non-observable ones. The aim is to estimate the coordinates and velocities of the UAV on the basis of linear observation Equation (6) and the motion model Equation (2). Therefore, the measurement vector has the following form:

(7) $$\begin{array}{c} m_{k}=\left(\begin{array}{c} m_{k}^{\phi }\\ m_{k}^{\lambda } \end{array}\right)=\left(\begin{array}{c} Y\left(t_{k}\right)\cos\phi_{i}\left(t_{k}\right)-X\left(t_{k}\right)\sin\phi_{i}\left(t_{k}\right)-\varepsilon_{k}^{y}\cos\phi_{i}\left(t_{k}\right)+\varepsilon_{k}^{x}\sin\phi_{i}\left(t_{k}\right)\\ +\varepsilon_{k}^{\phi }\left(X_{i}+\varepsilon_{k}^{x}-X\left(t_{k}\right)\right)\cos\phi_{i}\left(t_{k}\right)\\ Z\left(t_{k}\right)\sin\phi_{i}\left(t_{k}\right)\cos\lambda_{i}\left(t_{k}\right)-Y\left(t_{k}\right)\sin\lambda_{i}\left(t_{k}\right)-\varepsilon_{k}^{z}\sin\phi_{i}\left(t_{k}\right)\cos\lambda_{i}\left(t_{k}\right)\\ +\varepsilon_{k}^{y}\sin\lambda_{i}\left(t_{k}\right)+\varepsilon_{k}^{\lambda }\left(Y_{i}+\varepsilon_{k}^{y}-Y\left(t_{k}\right)\right)\cos\lambda_{i}\left(t_{k}\right) \end{array}\right) \end{array}$$

Thereby, we obtain the system Equation (7) of linear measurement equations, though the noise variance depends on non-observable coordinates. By using V.S. Pugachev’s method [15], one can obtain the unbiased estimate and the variance with the aid of a prediction-correction filter [24]. Moreover, we do not need to assume the Gaussian distribution of errors that is not valid in bearing observations with optical-electronic cameras with discrete image sensors.

### 4.2. Prediction-Correction Estimation

Assume that at the moment $t_{k}$, we have unbiased estimates $\hat{X}\left(t_{k}\right)$, such that:

(8) $$\begin{array}{c} E\left(\hat{X}\left(t_{k}\right)\right)=X\left(t_{k}\right) \end{array}$$

with the following matrix of the mean-square errors:

(9) $$\begin{array}{c} \hat{P}\left(t_{k}\right)=E\left\{\left(\hat{X}\left(t_{k}\right)-X\left(t_{k}\right)\right){\left(\hat{X}\left(t_{k}\right)-X\left(t_{k}\right)\right)}^{T}\right\}\\ =\left(\begin{array}{cccccc} {\hat{P}}^{xx}\left(t_{k}\right) & {\hat{P}}^{xy}\left(t_{k}\right) & ... & ... & ... & {\hat{P}}^{xV_{z}}\left(t_{k}\right)\\ {\hat{P}}^{xy}\left(t_{k}\right) & {\hat{P}}^{yy}\left(t_{k}\right) & ... & ... & ... & {\hat{P}}^{yV_{z}}\left(t_{k}\right)\\ {\hat{P}}^{xz}\left(t_{k}\right) & {\hat{P}}^{yz}\left(t_{k}\right) & ... & ... & ... & {\hat{P}}^{zV_{z}}\left(t_{k}\right)\\ {\hat{P}}^{xV_{x}}\left(t_{k}\right) & {\hat{P}}^{yV_{x}}\left(t_{k}\right) & ... & ... & ... & {\hat{P}}^{V_{x}V_{z}}\left(t_{k}\right)\\ {\hat{P}}^{xV_{y}}\left(t_{k}\right) & {\hat{P}}^{yV_{y}}\left(t_{k}\right) & ... & ... & ... & {\hat{P}}^{V_{y}V_{z}}\left(t_{k}\right)\\ {\hat{P}}^{xV_{z}}\left(t_{k}\right) & {\hat{P}}^{yV_{z}}\left(t_{k}\right) & ... & ... & ... & {\hat{P}}^{V_{z}V_{z}}\left(t_{k}\right) \end{array}\right) \end{array}$$

**Problem 1.**  *Find the unbiased estimates $\hat{X}\left(t_{k+1}\right)$ and matrix $\hat{P}\left(t_{k+1}\right)$ on the basis of estimates at the moment $t_{k},$$m_{k}$, the known position of the i-th observable object $\left(X_{i},Y_{i},Z_{i}\right)$ and the UAV’s motion parameter Equation (2). These estimates must satisfy Equation (8) and give the matrix Equation (9) for the moment $t_{k+1}$.*

#### 4.2.1. Prediction

The prediction is obtained by assuming that at the moment $t_{k+1}$, the values of $\phi (t_{k+1}),$ $\lambda (t_{k+1})$ will be known:

(10) $$\begin{array}{c} \tilde{X}\left(t_{k+1}\right)=F\hat{X}\left(t_{k}\right)+Ba\left(t_{k}\right)\\ {\tilde{m}}_{k+1}=\left(\begin{array}{c} {\tilde{m}}_{k+1}^{\phi }\\ {\tilde{m}}_{k+1}^{\lambda }\; \end{array}\right)=\left(\begin{array}{c} I\left(t_{k+1}\right)\left(\tilde{Y}\left(t_{k+1}\right)\cos\phi (t_{k+1})-\tilde{X}\left(t_{k+1}\right)\sin\phi (t_{k+1})\right)\\ I\left(t_{k+1}\right)\left(\tilde{Z}\left(t_{k+1}\right)\sin\phi (t_{k+1})\cos\lambda (t_{k+1})-\tilde{Y}\left(t_{k+1}\right)\sin\lambda (t_{k+1})\right) \end{array}\right) \end{array}$$

Assuming that the motion perturbations and the UAV position are uncorrelated, we obtain:

(11) $$\begin{array}{c} \tilde{P}\left(t_{k+1}\right)=\left(\begin{array}{cccccc} {\tilde{P}}^{xx}\left(t_{k+1}\right) & {\tilde{P}}^{xy}\left(t_{k+1}\right) & ... & ... & ... & {\tilde{P}}^{xV_{z}}\left(t_{k+1}\right)\\ {\tilde{P}}^{xy}\left(t_{k+1}\right) & {\tilde{P}}^{yy}\left(t_{k+1}\right) & ... & ... & ... & {\tilde{P}}^{yV_{z}}\left(t_{k+1}\right)\\ {\tilde{P}}^{xz}\left(t_{k+1}\right) & {\tilde{P}}^{yz}\left(t_{k+1}\right) & ... & ... & ... & {\tilde{P}}^{zV_{z}}\left(t_{k+1}\right)\\ {\tilde{P}}^{xV_{x}}\left(t_{k+1}\right) & {\tilde{P}}^{yV_{x}}\left(t_{k+1}\right) & ... & ... & ... & {\tilde{P}}^{V_{x}V_{z}}\left(t_{k+1}\right)\\ {\tilde{P}}^{xV_{y}}\left(t_{k+1}\right) & {\tilde{P}}^{yV_{y}}\left(t_{k+1}\right) & ... & ... & ... & {\tilde{P}}^{V_{y}V_{z}}\left(t_{k+1}\right)\\ {\tilde{P}}^{xV_{z}}\left(t_{k+1}\right) & {\tilde{P}}^{yV_{z}}\left(t_{k+1}\right) & ... & ... & ... & {\tilde{P}}^{V_{z}V_{z}}\left(t_{k+1}\right) \end{array}\right) \end{array}$$

where the elements of this matrix are:

(12) $$\begin{array}{c} {\tilde{P}}^{xx}\left(t_{k+1}\right)={\hat{P}}^{xx}\left(t_{k}\right)+2{\hat{P}}^{xV_{x}}\left(t_{k}\right)\Delta t+{\hat{P}}^{V_{x}V_{x}}\left(t_{k}\right)\Delta t^{2}\\ {\tilde{P}}^{yy}\left(t_{k+1}\right)={\hat{P}}^{yy}\left(t_{k}\right)+2{\hat{P}}^{yV_{y}}\left(t_{k}\right)\Delta t+{\hat{P}}^{V_{y}V_{y}}\left(t_{k}\right)\Delta t^{2}\\ {\tilde{P}}^{zz}\left(t_{k+1}\right)={\hat{P}}^{zz}\left(t_{k}\right)+2{\hat{P}}^{zV_{z}}\left(t_{k}\right)\Delta t+{\hat{P}}^{V_{z}V_{z}}\left(t_{k}\right)\Delta t^{2}\\ {\tilde{P}}^{xy}\left(t_{k+1}\right)={\hat{P}}^{xy}\left(t_{k}\right)+{\hat{P}}^{xV_{y}}\left(t_{k}\right)\Delta t+{\hat{P}}^{yV_{x}}\left(t_{k}\right)\Delta t+{\hat{P}}^{V_{x}V_{y}}\left(t_{k}\right)\Delta t^{2}\\ {\tilde{P}}^{xz}\left(t_{k+1}\right)={\hat{P}}^{xz}\left(t_{k}\right)+{\hat{P}}^{xV_{z}}\left(t_{k}\right)\Delta t+{\hat{P}}^{zV_{x}}\left(t_{k}\right)\Delta t+{\hat{P}}^{V_{x}V_{z}}\left(t_{k}\right)\Delta t^{2}\\ {\tilde{P}}^{yz}\left(t_{k+1}\right)={\hat{P}}^{yz}\left(t_{k}\right)+{\hat{P}}^{yV_{z}}\left(t_{k}\right)\Delta t+{\hat{P}}^{zV_{y}}\left(t_{k}\right)\Delta t+{\hat{P}}^{V_{y}V_{z}}\left(t_{k}\right)\Delta t^{2}\\ {\tilde{P}}^{xV_{x}}\left(t_{k+1}\right)={\hat{P}}^{xV_{x}}\left(t_{k}\right)+{\hat{P}}^{V_{x}V_{x}}\left(t_{k}\right)\Delta t\\ {\tilde{P}}^{xV_{y}}\left(t_{k+1}\right)={\hat{P}}^{xV_{y}}\left(t_{k}\right)+{\hat{P}}^{V_{x}V_{y}}\left(t_{k}\right)\Delta t\\ {\tilde{P}}^{xV_{z}}\left(t_{k+1}\right)={\hat{P}}^{xV_{z}}\left(t_{k}\right)+{\hat{P}}^{V_{x}V_{z}}\left(t_{k}\right)\Delta t\\ {\tilde{P}}^{yV_{x}}\left(t_{k+1}\right)={\hat{P}}^{yV_{x}}\left(t_{k}\right)+{\hat{P}}^{V_{x}V_{y}}\left(t_{k}\right)\Delta t\\ {\tilde{P}}^{yV_{y}}\left(t_{k+1}\right)={\hat{P}}^{yV_{y}}\left(t_{k}\right)+{\hat{P}}^{V_{y}V_{y}}\left(t_{k}\right)\Delta t\\ {\tilde{P}}^{yV_{z}}\left(t_{k+1}\right)={\hat{P}}^{yV_{z}}\left(t_{k}\right)+{\hat{P}}^{V_{y}V_{z}}\left(t_{k}\right)\Delta t\\ {\tilde{P}}^{zV_{x}}\left(t_{k+1}\right)={\hat{P}}^{zV_{x}}\left(t_{k}\right)+{\hat{P}}^{V_{x}V_{z}}\left(t_{k}\right)\Delta t\\ {\tilde{P}}^{zV_{y}}\left(t_{k+1}\right)={\hat{P}}^{zV_{y}}\left(t_{k}\right)+{\hat{P}}^{V_{y}V_{z}}\left(t_{k}\right)\Delta t\\ {\tilde{P}}^{zV_{z}}\left(t_{k+1}\right)={\hat{P}}^{zV_{z}}\left(t_{k}\right)+{\hat{P}}^{V_{z}V_{z}}\left(t_{k}\right)\Delta t\\ {\tilde{P}}^{V_{x}V_{x}}\left(t_{k+1}\right)={\hat{P}}^{V_{x}V_{x}}\left(t_{k}\right)+\sigma_{X}^{2}\\ {\tilde{P}}^{V_{y}V_{y}}\left(t_{k+1}\right)={\hat{P}}^{V_{y}V_{y}}\left(t_{k}\right)+\sigma_{Y}^{2}\\ {\tilde{P}}^{V_{z}V_{z}}\left(t_{k+1}\right)={\hat{P}}^{V_{z}V_{z}}\left(t_{k}\right)+\sigma_{Z}^{2}\\ {\tilde{P}}^{V_{x}V_{y}}\left(t_{k+1}\right)={\hat{P}}^{V_{x}V_{y}}\left(t_{k}\right)\\ {\tilde{P}}^{V_{x}V_{z}}\left(t_{k+1}\right)={\hat{P}}^{V_{x}V_{z}}\left(t_{k}\right)\\ {\tilde{P}}^{V_{y}V_{z}}\left(t_{k+1}\right)={\hat{P}}^{V_{y}V_{z}}\left(t_{k}\right) \end{array}$$

Note that $\sigma_{X}^{2}$ is not the same as $\sigma_{x}^{2}$ and similarly for the other indices.

Then, the following values ${\tilde{P}}^{xm}\left(t_{k+1}\right),$ ${\tilde{P}}^{ym}\left(t_{k+1}\right),$ ${\tilde{P}}^{zm}\left(t_{k+1}\right),$ ${\tilde{P}}^{V_{x}m}\left(t_{k+1}\right),$ ${\tilde{P}}^{V_{y}m}\left(t_{k+1}\right),$ ${\tilde{P}}^{V_{z}m}\left(t_{k+1}\right),$ ${\tilde{P}}^{mm}\left(t_{k+1}\right)$ should be calculated using the following relations:

$$\begin{array}{c} \left(\begin{array}{c} m_{k+1}^{\phi }-{\tilde{m}}_{k+1}^{\phi }\\ m_{k+1}^{\lambda }-{\tilde{m}}_{k+1}^{\lambda } \end{array}\right)\\ =\left(\begin{array}{c} \left(Y\left(t_{k+1}\right)-\tilde{Y}\left(t_{k+1}\right)\right)\cos\phi_{i}\left(t_{k+1}\right)-\left(X\left(t_{k+1}\right)-\tilde{X}\left(t_{k+1}\right)\right)\sin\phi_{i}\left(t_{k+1}\right)\\ -\varepsilon_{k+1}^{y}\cos\phi_{i}\left(t_{k+1}\right)+\varepsilon_{k+1}^{x}\sin\phi_{i}\left(t_{k+1}\right)+\varepsilon_{k+1}^{\phi }\left(X_{i}+\varepsilon_{k+1}^{x}-X\left(t_{k+1}\right)\right)\cos\phi_{i}\left(t_{k+1}\right)\\ \left(Z\left(t_{k+1}\right)-\tilde{Z}\left(t_{k+1}\right)\right)\sin\phi_{i}\left(t_{k+1}\right)\cos\lambda_{i}\left(t_{k+1}\right)-\left(Y\left(t_{k+1}\right)-\tilde{Y}\left(t_{k+1}\right)\right)\sin\lambda_{i}\left(t_{k+1}\right)\\ -\varepsilon_{k+1}^{z}\sin\phi_{i}\left(t_{k+1}\right)\cos\lambda_{i}\left(t_{k+1}\right)+\varepsilon_{k+1}^{y}\sin\lambda_{i}\left(t_{k+1}\right)+\varepsilon_{k+1}^{\lambda }\left(Y_{i}+\varepsilon_{k+1}^{y}-Y\left(t_{k+1}\right)\right)\cos\lambda_{i}\left(t_{k+1}\right) \end{array}\right) \end{array}$$

and the identities:

$$\begin{array}{cc} & X_{i}-X\left(t_{k+1}\right)=X_{i}-\tilde{X}\left(t_{k+1}\right)-\left(X\left(t_{k+1}\right)-\tilde{X}\left(t_{k+1}\right)\right)\\ & Y_{i}-Y\left(t_{k+1}\right)=Y_{i}-\tilde{Y}\left(t_{k+1}\right)-\left(Y\left(t_{k+1}\right)-\tilde{Y}\left(t_{k+1}\right)\right)\\ & Z_{i}-Z\left(t_{k+1}\right)=Z_{i}-\tilde{Z}\left(t_{k+1}\right)-\left(Z\left(t_{k+1}\right)-\tilde{Z}\left(t_{k+1}\right)\right) \end{array}$$

where we consider that the position of the *i*-th object is known and use the non-correlatedness of $\varepsilon_{k+1}^{x},$ $\varepsilon_{k+1}^{y},$ $\varepsilon_{k+1}^{z},$ $\varepsilon_{k+1}^{\phi },$ $\varepsilon_{k+1}^{\lambda }$ and differences $(X\left(t_{k+1}\right)-\tilde{X}\left(t_{k+1}\right)),$ $\left(Y\left(t_{k+1}\right)-\tilde{Y}\left(t_{k+1}\right)\right)$ and $(Z\left(t_{k+1}\right)-\tilde{Z}\left(t_{k+1}\right)).$

Finally, we get:

(13) $$\begin{array}{c} {\left[{\tilde{P}}^{xm}\left(t_{k+1}\right)\right]}^{T}=E\left[\left(X\left(t_{k+1}\right)-\tilde{X}\left(t_{k+1}\right)\right)\left(\begin{array}{c} m_{k+1}^{\phi }-{\tilde{m}}_{k+1}^{\phi }\\ m_{k+1}^{\lambda }-{\tilde{m}}_{k+1}^{\lambda } \end{array}\right)\right]\\ =\left(\begin{array}{c} {\tilde{P}}^{xy}\left(t_{k+1}\right)\cos\phi_{i}\left(t_{k+1}\right)-{\tilde{P}}^{xx}\left(t_{k+1}\right)\sin\phi_{i}\left(t_{k+1}\right)\\ {\tilde{P}}^{xz}\left(t_{k+1}\right)\sin\phi_{i}\left(t_{k+1}\right)\cos\lambda_{i}\left(t_{k+1}\right)-{\tilde{P}}^{xy}\left(t_{k+1}\right)\sin\lambda_{i}\left(t_{k+1}\right) \end{array}\right), \end{array}$$

(14) $$\begin{array}{c} {\left[{\tilde{P}}^{ym}\left(t_{k+1}\right)\right]}^{T}=E\left[\left(Y\left(t_{k+1}\right)-\tilde{Y}\left(t_{k+1}\right)\right)\left(\begin{array}{c} m_{k+1}^{\phi }-{\tilde{m}}_{k+1}^{\phi }\\ m_{k+1}^{\lambda }-{\tilde{m}}_{k+1}^{\lambda } \end{array}\right)\right]\\ =\left(\begin{array}{c} {\tilde{P}}^{yy}\left(t_{k+1}\right)\cos\phi_{i}\left(t_{k+1}\right)-{\tilde{P}}^{xy}\left(t_{k+1}\right)\sin\phi_{i}\left(t_{k+1}\right)\\ {\tilde{P}}^{yz}\left(t_{k+1}\right)\sin\phi_{i}\left(t_{k+1}\right)\cos\lambda_{i}\left(t_{k+1}\right)-{\tilde{P}}^{yy}\left(t_{k+1}\right)\sin\lambda_{i}\left(t_{k+1}\right) \end{array}\right), \end{array}$$

(15) $$\begin{array}{c} {\left[{\tilde{P}}^{zm}\left(t_{k+1}\right)\right]}^{T}=E\left[\left(Z\left(t_{k+1}\right)-\tilde{Z}\left(t_{k+1}\right)\right)\left(\begin{array}{c} m_{k+1}^{\phi }-{\tilde{m}}_{k+1}^{\phi }\\ m_{k+1}^{\lambda }-{\tilde{m}}_{k+1}^{\lambda } \end{array}\right)\right]\\ =\left(\begin{array}{c} {\tilde{P}}^{yz}\left(t_{k+1}\right)\cos\phi_{i}\left(t_{k+1}\right)-{\tilde{P}}^{xz}\left(t_{k+1}\right)\sin\phi_{i}\left(t_{k+1}\right)\\ {\tilde{P}}^{zz}\left(t_{k+1}\right)\sin\phi_{i}\left(t_{k+1}\right)\cos\lambda_{i}\left(t_{k+1}\right)-{\tilde{P}}^{yz}\left(t_{k+1}\right)\sin\lambda_{i}\left(t_{k+1}\right) \end{array}\right), \end{array}$$

(16) $$\begin{array}{c} {\left[{\tilde{P}}^{V_{x}m}\left(t_{k+1}\right)\right]}^{T}=E\left[\left(V_{x}\left(t_{k+1}\right)-{\tilde{V}}_{x}\left(t_{k+1}\right)\right)\left(\begin{array}{c} m_{k+1}^{\phi }-{\tilde{m}}_{k+1}^{\phi }\\ m_{k+1}^{\lambda }-{\tilde{m}}_{k+1}^{\lambda } \end{array}\right)\right]\\ =\left(\begin{array}{c} {\tilde{P}}^{yV_{x}}\left(t_{k+1}\right)\cos\phi_{i}\left(t_{k+1}\right)-{\tilde{P}}^{xV_{x}}\left(t_{k+1}\right)\sin\phi_{i}\left(t_{k+1}\right)\\ {\tilde{P}}^{zV_{x}}\left(t_{k+1}\right)\sin\phi_{i}\left(t_{k+1}\right)\cos\lambda_{i}\left(t_{k+1}\right)-{\tilde{P}}^{yV_{x}}\left(t_{k+1}\right)\sin\lambda_{i}\left(t_{k+1}\right) \end{array}\right), \end{array}$$

(17) $$\begin{array}{c} {\left[{\tilde{P}}^{V_{y}m}\left(t_{k+1}\right)\right]}^{T}=E\left[\left(V_{y}\left(t_{k+1}\right)-{\tilde{V}}_{y}\left(t_{k+1}\right)\right)\left(\begin{array}{c} m_{k+1}^{\phi }-{\tilde{m}}_{k+1}^{\phi }\\ m_{k+1}^{\lambda }-{\tilde{m}}_{k+1}^{\lambda } \end{array}\right)\right]\\ =\left(\begin{array}{c} {\tilde{P}}^{yV_{y}}\left(t_{k+1}\right)\cos\phi_{i}\left(t_{k+1}\right)-{\tilde{P}}^{xV_{y}}\left(t_{k+1}\right)\sin\phi_{i}\left(t_{k+1}\right)\\ {\tilde{P}}^{zV_{y}}\left(t_{k+1}\right)\sin\phi_{i}\left(t_{k+1}\right)\cos\lambda_{i}\left(t_{k+1}\right)-{\tilde{P}}^{yV_{y}}\left(t_{k+1}\right)\sin\lambda_{i}\left(t_{k+1}\right) \end{array}\right), \end{array}$$

(18) $$\begin{array}{c} {\left[{\tilde{P}}^{V_{z}m}\left(t_{k+1}\right)\right]}^{T}=E\left[\left(V_{z}\left(t_{k+1}\right)-{\tilde{V}}_{z}\left(t_{k+1}\right)\right)\left(\begin{array}{c} m_{k+1}^{\phi }-{\tilde{m}}_{k+1}^{\phi }\\ m_{k+1}^{\lambda }-{\tilde{m}}_{k+1}^{\lambda } \end{array}\right)\right]\\ =\left(\begin{array}{c} {\tilde{P}}^{yV_{z}}\left(t_{k+1}\right)\cos\phi_{i}\left(t_{k+1}\right)-{\tilde{P}}^{xV_{z}}\left(t_{k+1}\right)\sin\phi_{i}\left(t_{k+1}\right)\\ {\tilde{P}}^{zV_{z}}\left(t_{k+1}\right)\sin\phi_{i}\left(t_{k+1}\right)\cos\lambda_{i}\left(t_{k+1}\right)-{\tilde{P}}^{yV_{z}}\left(t_{k+1}\right)\sin\lambda_{i}\left(t_{k+1}\right) \end{array}\right). \end{array}$$

In a similar way, we calculate:

(19) $$\begin{array}{c} {\left({\tilde{P}}^{mm}\left(t_{k+1}\right)\right)}^{-1}\\ ={\left[E\left(\begin{array}{cc} {\left(m_{k+1}^{\phi }-{\tilde{m}}_{k+1}^{\phi }\right)}^{2} & \left(m_{k+1}^{\phi }-{\tilde{m}}_{k+1}^{\phi }\right)\left(m_{k+1}^{\lambda }-{\tilde{m}}_{k+1}^{\lambda }\right)\\ \left(m_{k+1}^{\phi }-{\tilde{m}}_{k+1}^{\phi }\right)\left(m_{k+1}^{\lambda }-{\tilde{m}}_{k+1}^{\lambda }\right) & {\left(m_{k+1}^{\lambda }-{\tilde{m}}_{k+1}^{\lambda }\right)}^{2} \end{array}\right)\right]}^{-1} \end{array}$$

Therefore:

$$\begin{array}{c} E\left[{\left(m_{k+1}^{\phi }-{\tilde{m}}_{k+1}^{\phi }\right)}^{2}\right]\\ ={\tilde{P}}^{yy}\left(t_{k+1}\right)\cos^{2}\phi_{i}\left(t_{k+1}\right)-{\tilde{P}}^{xy}\left(t_{k+1}\right)\sin2\phi_{i}\left(t_{k+1}\right)\\ +{\tilde{P}}^{xx}\left(t_{k+1}\right)\sin^{2}\phi_{i}\left(t_{k+1}\right)+\sigma_{y}^{2}\cos^{2}\phi_{i}\left(t_{k+1}\right)+\sigma_{x}^{2}\sin^{2}\phi_{i}\left(t_{k+1}\right)+\sigma_{\phi }^{2}({\left(X_{i}-\hat{X}\left(t_{k}\right)\right)}^{2}\\ +{\tilde{P}}^{xx}\left(t_{k+1}\right)+\sigma_{x}^{2})\cos^{2}\phi_{i}\left(t_{k+1}\right)\\ E\left[\left(m_{k+1}^{\phi }-{\tilde{m}}_{k+1}^{\phi }\right)\left(m_{k+1}^{\lambda }-{\tilde{m}}_{k+1}^{\lambda }\right)\right]\\ ={\tilde{P}}^{yz}\left(t_{k+1}\right)\sin\phi_{i}\left(t_{k+1}\right)\cos\phi_{i}\left(t_{k+1}\right)\cos\lambda_{i}\left(t_{k+1}\right)\\ -{\tilde{P}}^{yy}\left(t_{k+1}\right)\cos\phi_{i}\left(t_{k+1}\right)\sin\lambda_{i}\left(t_{k+1}\right)-{\tilde{P}}^{xz}\left(t_{k+1}\right)\sin^{2}\phi_{i}\left(t_{k+1}\right)\cos\lambda_{i}\left(t_{k+1}\right)\\ +{\tilde{P}}^{xy}\left(t_{k+1}\right)\sin\phi_{i}\left(t_{k+1}\right)\sin\lambda_{i}\left(t_{k+1}\right)\\ E[{\left(m_{k+1}^{\lambda }-{\tilde{m}}_{k+1}^{\lambda }\right)}^{2}]\\ ={\tilde{P}}^{zz}\left(t_{k+1}\right)\sin^{2}\phi_{i}\left(t_{k+1}\right)\cos^{2}\lambda_{i}\left(t_{k+1}\right)-{\tilde{P}}^{yz}\left(t_{k+1}\right)\sin\phi_{i}\left(t_{k+1}\right)\sin2\lambda_{i}\left(t_{k+1}\right)\\ +{\tilde{P}}^{yy}\left(t_{k+1}\right)\sin^{2}\lambda_{i}\left(t_{k+1}\right)+\sigma_{z}^{2}\sin^{2}\phi_{i}\left(t_{k+1}\right)\cos^{2}\lambda_{i}\left(t_{k+1}\right)+\sigma_{y}^{2}\sin^{2}\lambda_{i}\left(t_{k+1}\right)+\sigma_{\lambda }^{2}({\left(Y_{i}-\hat{Y}\left(t_{k}\right)\right)}^{2}\\ +{\tilde{P}}^{yy}\left(t_{k+1}\right)+\sigma_{y}^{2})\cos^{2}\lambda_{i}\left(t_{k+1}\right) \end{array}$$

#### 4.2.2. Correction

After getting the measurements at the moment $t_{k+1}$, one can obtain the estimate of the UAV position at this moment. Therefore, the solution of Problem 1 has the form:

(20) $$\begin{array}{c} \hat{X}\left(t_{k+1}\right)=\tilde{X}\left(t_{k+1}\right)+\tilde{P}\left(t_{k+1}\right){\left({\tilde{P}}^{mm}\left(t_{k+1}\right)\right)}^{-1}\left(m_{k+1}-{\tilde{m}}_{k+1}\right) \end{array}$$

and the matrix of the mean square errors is equal to:

(21) $$\begin{array}{c} \hat{P}\left(t_{k+1}\right)=\tilde{P}\left(t_{k+1}\right)-\tilde{P}\left(t_{k+1}\right){\left({\tilde{P}}^{mm}\left(t_{k+1}\right)\right)}^{-1}\tilde{P}{\left(t_{k+1}\right)}^{T} \end{array}$$

where:

$$\tilde{P}\left(t_{k+1}\right)={\left({\tilde{P}}^{xm}\left(t_{k+1}\right),{\tilde{P}}^{ym}\left(t_{k+1}\right),...,{\tilde{P}}^{V_{z}m}\left(t_{k+1}\right)\right)}^{T}$$

## 5. Robust Filtering on the Basis of the UAV Motion Model

The RANSAC method calculates the rotation matrix and the coordinates of the camera $\left\{A^{*},b^{*}\right\}$ in the Earth coordinate system with some minor error. However, the RANSAC method can give quite the wrong answer, called an outlier. It could happen, for example, if the frames $I_{c}$ and $I_{m}$ do not depict common objects. We provide further a method that makes a decision about whether $\left\{A^{*},b^{*}\right\}$ is an outlier or not. This problem has been considered in relation to the exclusion of outliers in the RANSAC-type procedures [25,26]. Here, we use the modification of the robust RANSAC [23] based on PKF for bearing-only observations [17].

After the prediction step of the Kalman filter, one can estimate the UAV (camera) position and the matrix of the mean square errors:

$$\tilde{X}=\tilde{X}\left(t_{k+1}\right),\;\;\;\tilde{P}=\tilde{P}\left(t_{k+1}\right).$$

Like in [26], one can suppose that the corresponding probability density is Gaussian. The reason is that the PKF gives the best linear estimates obtained until the current time t. This estimate is the sum of uncorrelated random variables with almost the same variations, at least on the short intervals preceding the current time. It gives the opportunity to approximate the probability density distribution by the Gaussian one. Further extension of the robust RANSAC technique is based on the prior distribution of the UAV attitude. The approach has been developed in [23,27] on the basis of the extended Kalman filter (EKF). However, the estimate given by the EKF has an unknown bias and, of course, does not give the posterior Gaussian distribution. Therefore, the PKF, which gives an unbiased estimate, looks more preferable under the hypothesis of the posterior Gaussian distribution. Therefore, at the (k+1)-th step, the posterior distribution of $r_{i}^{{}^{\prime }}$ corresponding to an inlier is assumed to be Gaussian, that is according to Equation (1):

$$N(A_{k}\left(r_{i}-\tilde{X}\left(t_{k+1}\right)\right),A_{k}\left(\tilde{P}\left(t_{k+1}\right)+P_{rr}\right)A_{k}^{T})$$

where $P_{rr}$ is the covariance matrix of the landmarks localization. Further, in the estimation algorithm, the pair $\left\{A_{k},\tilde{X}\left(t_{k+1}\right)\right\}$ is considered as an outlier at the confidence level 95% if:

$$∥r_{i}^{{}^{\prime }}-A_{k}\left(r_{i}-\tilde{X}\left(t_{k+1}\right)\right)∥\geq 2*Sp\left[A_{k}\left(\tilde{P}\left(t_{k+1}\right)+P_{rr}\right)A_{k}^{T}\right]$$

**Figure 4:**
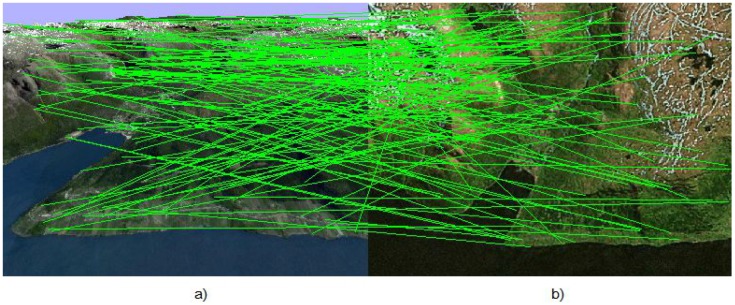
Correspondence between (**a**) (image $I_{c}$ of the on-board camera of the UAV) and (**b**) (template image loaded in the UAV memory in advance) feature points found without the UAV motion model. One can observe chaotic correspondence, which gives a huge number of outliers.

**Figure 5:**
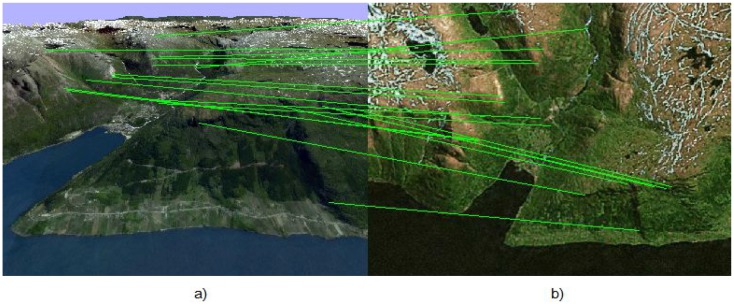
Correspondence between (**a**) (image $I_{c}$ of the on-board camera of the UAV) and (**b**) (template image loaded in the UAV memory in advance) feature points found with the aid of RANSAC based on the UAV motion model. The number of outliers reduces substantially.

Otherwise, the correction step is based on $\{A_{k},\tilde{X}\left(t_{k+1}\right)\}.$ Of course, all such nonlinear conjectures need confirmation on the basis of statistical modeling, which is one of the results of this article. One can observe the performance of robust filtering in the following figures. Figure 4 shows the correspondence between feature points obtained without a UAV motion model. Next, Figure 5 shows the correspondence established on the basis of the UAV motion model. The number of outliers reduces substantially.

## 6. Control of the UAV

Control of a UAV that ensures its motion along the reference trajectory may be determined on the basis of the standard deterministic linear-quadratic approach [28]. However, the problem of control on the basis of bearing-only observation is different from the standard one. It should be underlined that this problem is a non-linear one and cannot be solved by the standard way. The problem of the optimal control for the system Equation (2) is the stochastic one with incomplete information and does not have an explicit solution. However, for practical reasons, one can simplify it by considering the locally optimal control. Here, we discuss the following problem:

**Problem 2.**  Find the locally optimal controls $a_{x}\left(t_{k}\right),$$a_{y}\left(t_{k}\right)$ and $a_{z}\left(t_{k}\right)$ aimed to keep the motion of the UAV along the reference trajectory.

Assume that we have some reference trajectory $X_{nom}\left(t_{k}\right).$

Therefore, at the moment $t_{k+1}$, we have to minimize the following expressions:

$$\begin{array}{cc} & E_{1}=E{\left\{{\left(X\left(t_{k+1}\right)-X_{nom}\left(t_{k+1}\right)\right)}^{2}+\left(V_{x}\left(t_{k+1}\right)-V_{x_{nom}}\left(t_{k+1}\right)\right)\Delta t\right)}^{2}\}\to \min_{a_{x}\left(t_{k}\right)}\\ & E_{2}=E{\left\{{\left(Y\left(t_{k+1}\right)-Y_{nom}\left(t_{k+1}\right)\right)}^{2}+\left(V_{z}\left(t_{k+1}\right)-V_{z_{nom}}\left(t_{k+1}\right)\right)\Delta t\right)}^{2}\}\to \min_{a_{y}\left(t_{k}\right)}\\ & E_{3}=E{\left\{{\left(Z\left(t_{k+1}\right)-Z_{nom}\left(t_{k+1}\right)\right)}^{2}+\left(V_{z}\left(t_{k+1}\right)-V_{z_{nom}}\left(t_{k+1}\right)\right)\Delta t\right)}^{2}\}\to \min_{a_{z}\left(t_{k}\right)} \end{array}$$

Let us consider the components of the $E_{1}$ expression:

$$\begin{array}{cc} & X\left(t_{k+1}\right)-X_{nom}\left(t_{k+1}\right)=X\left(t_{k}\right)-\hat{X}\left(t_{k}\right)-\left(X_{nom}\left(t_{k}\right)-\hat{X}\left(t_{k}\right)\right)\\ & +\left(V_{x}\left(t_{k}\right)-{\hat{V}}_{x}\left(t_{k}\right)\right)\Delta t-\left(V_{x_{nom}}\left(t_{k}\right)-{\hat{V}}_{x}\left(t_{k}\right)\right)\Delta t+\left(a_{x}\left(t_{k}\right)-a_{x_{nom}}\left(t_{k}\right)\right)\frac{\Delta t^{2}}{2}\\ & V_{x}\left(t_{k+1}\right)-V_{x_{nom}}\left(t_{k+1}\right)=V_{x}\left(t_{k}\right)-{\hat{V}}_{x}\left(t_{k}\right)-\left(V_{x_{nom}}\left(t_{k}\right)-{\hat{V}}_{x}\left(t_{k}\right)\right)+\left(a_{x}\left(t_{k}\right)-a_{x_{nom}}\left(t_{k}\right)\right)\Delta t\\ & +W_{x}\left(t_{k}\right) \end{array}$$

Then, we square these components and take the derivative of the sum with respect to $a_{x}\left(t_{k}\right)$ given that some components are uncorrelated:

$$\begin{array}{cc} & E\{\left(X\left(t_{k}\right)-\hat{X}\left(t_{k}\right)\right)\left(X_{nom}\left(t_{k}\right)-\hat{X}\left(t_{k}\right)\right)\}=0\\ & E\{\left(V_{x}\left(t_{k}\right)-{\hat{V}}_{x}\left(t_{k}\right)\right)\left(V_{x_{nom}}\left(t_{k}\right)-{\hat{V}}_{x}\left(t_{k}\right)\right)\}=0 \end{array}$$

Finally, we get:

(22) $$\begin{array}{c} a_{x}\left(t_{k}\right)=a_{x_{nom}}\left(t_{k}\right)+\frac{2(X_{nom}\left(t_{k}\right)-\hat{X}\left(t_{k}\right))}{5\Delta t^{2}}+\frac{6(V_{x_{nom}}\left(t_{k}\right)-{\hat{V}}_{x}\left(t_{k}\right))}{5\Delta t} \end{array}$$

We take into account that the acceleration of the UAV has limitations $\left[a_{x_{min}},a_{x_{max}}\right]$, so the control acceleration has the form:

$$a_{x}^{c}\left(t_{k}\right)=\left\{\begin{array}{cc} a_{x_{min}} & if\;a_{x}\left(t_{k}\right)<a_{x_{min}},\\ a_{x}\left(t_{k}\right) & if\;a_{x_{min}}\leq a_{x}\left(t_{k}\right)\leq a_{x_{max}},\\ a_{x_{max}} & if\;a_{x}\left(t_{k}\right)>a_{x_{max}}. \end{array}\right.$$

Similarly, we obtain the expressions for $a_{y}^{c}\left(t_{k}\right)$ and $a_{z}^{c}\left(t_{k}\right).$ Thus, we get the following solution of Problem 2 [19]:

(23) $$\begin{array}{c} \hat{a}\left(t_{k}\right)={\hat{a}}_{nom}\left(t_{k}\right)+\frac{2}{5\Delta t^{2}}\left({\hat{X}}_{nom}\left(t_{k}\right)-\hat{X}\left(t_{k}\right)\right)+\frac{6}{5\Delta t}\left({\hat{V}}_{nom}\left(t_{k}\right)-\hat{V}\left(t_{k}\right)\right) \end{array}$$

where:

$$\begin{array}{c} \hat{a}\left(t_{k}\right)={\left(a_{x}\left(t_{k}\right),a_{y}\left(t_{k}\right),a_{z}\left(t_{k}\right)\right)}^{T}\\ \hat{X}\left(t_{k}\right)={\left(X\left(t_{k}\right),Y\left(t_{k}\right),Z\left(t_{k}\right)\right)}^{T}\\ \hat{V}\left(t_{k}\right)={\left(V_{x}\left(t_{k}\right),V_{y}\left(t_{k}\right),V_{z}\left(t_{k}\right)\right)}^{T} \end{array}$$

and similarly for the nominal trajectory components.

## 7. Experimental Results

**Figure 6:**
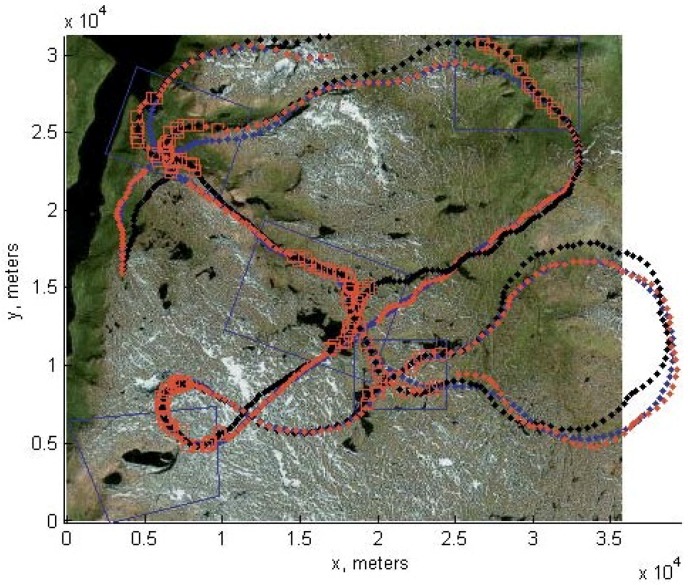
Blue dots corresponds to the reference trajectory and black dots to the real path. Blue squares show the localization of the terrain areas corresponding to the template images, and red squares show the moments where the estimates of the UAV positions have been obtained and assumed to be reliable according to the robust RANSAC algorithm described in Section 5.

In this section, we give the results of the algorithm’s modeling. The UAV is virtually flying over the landscape shown in Figure 1. This image has been obtained from Google Maps, and for verification of the algorithm, the image of the same region obtained from another Bing satellite was used. Therefore, these two images modeled the preliminary downloaded template and the image obtained by the on-board camera. The result of virtual flight experiment is shown in Figure 6.

## 8. Results and Discussion

**Figure 7:**
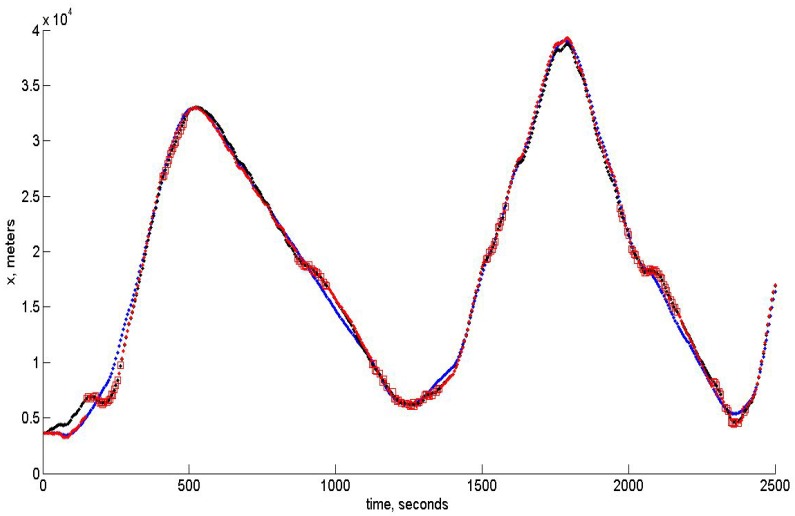
Tracking of the *x*-coordinate.

**Figure 8:**
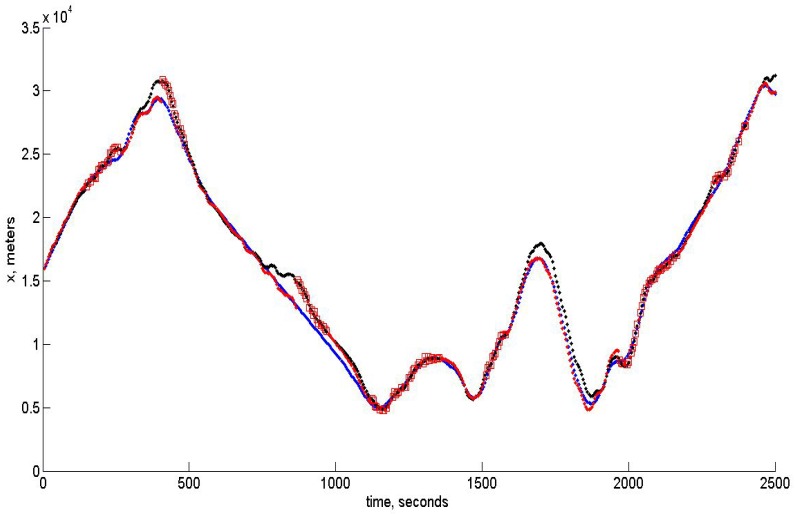
Tracking of the *y*-coordinate.

**Figure 9:**
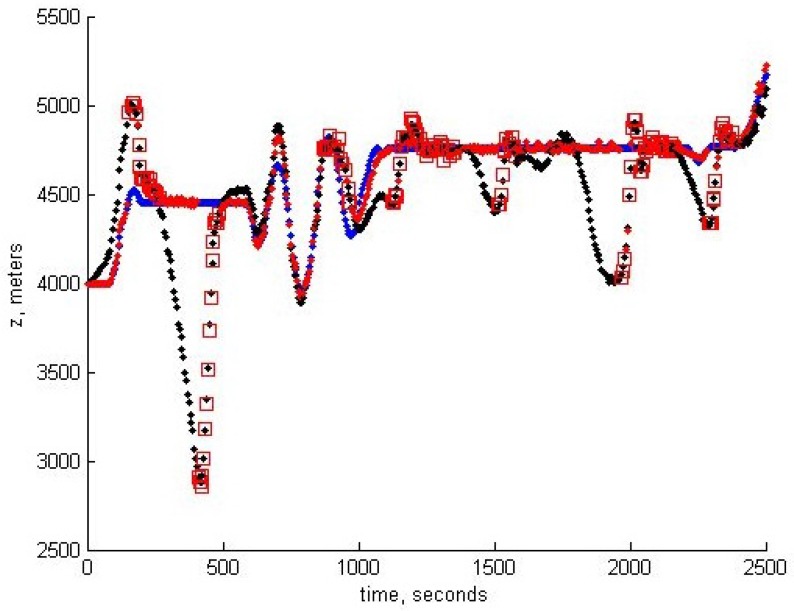
Tracking of the *z*-coordinate.

In the modeling of the control algorithm, we use the UAV moving approximately with a velocity of 50 m/s, though the change of the altitude is assumed to be rather substantial. The control algorithm takes into account the constraints imposed on linear acceleration and angular velocities. The software developed for modeling may be used in a real on-board navigation system, as well. Moreover, the filtering algorithm based on unbiased estimation may be easily incorporated with the INS, since it gives also the unbiased square error estimates, which opens the way to correct data fusion. The quality of tracking for x,y,z components is shown below in Figure 7, Figure 8 and Figure 9, respectively. In all of these figures, blue dots correspond to the reference trajectory, black dots to the real path and red squares to the moments, where the estimates of the UAV positions have been obtained and assumed to be reliable according to the robust RANSAC algorithm. One can observe that in the “measurement” areas, the algorithm estimates the coordinates with high accuracy, and the control provides the tracking with high accuracy, as well.

**Figure 10:**
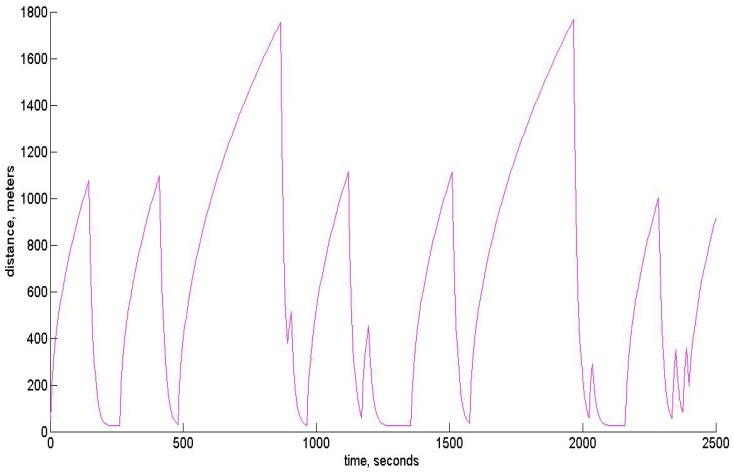
Averaged standard deviation of the position estimation error. The limit value in the observation areas is close to 24.5 m. One can see that in the areas of no observations, the SD monotonically increases.

The high accuracy is in accordance with the standard deviation (SD) theoretically calculated from the PKF. The value of the averaged standard deviation, which is the square root of $P_{xx}\left(t\right)+P_{yy}\left(t\right)+P_{zz}\left(t\right)$, is shown in Figure 10 below.

## 9. Conclusions

The main result of the work is the new algorithm of the UAV control based on the observation of the landmarks in a 3D environment. The new RANSAC based on the UAV motion model permits one to exclude the huge number of outliers and, by that, to provide the reliable set of data for the estimation of the UAV position on the basis of the novel non-biased PKF algorithm. This work is just the beginning of the implementation of this approach in the navigation of UAVs during long-term autonomous missions.
